# The Actin-Disassembly Protein Glia Maturation Factor γ Enhances Actin Remodeling and B Cell Antigen Receptor Signaling at the Immune Synapse

**DOI:** 10.3389/fcell.2021.647063

**Published:** 2021-07-09

**Authors:** Nikola Deretic, Madison Bolger-Munro, Kate Choi, Libin Abraham, Michael R. Gold

**Affiliations:** Department of Microbiology and Immunology, Life Sciences Institute, University of British Columbia, Vancouver, BC, Canada

**Keywords:** B cell, actin, B cell receptor, immune synapse, signal transduction, cell spreading, glia maturation factor-γ

## Abstract

Signaling by the B cell antigen receptor (BCR) initiates actin remodeling. The assembly of branched actin networks that are nucleated by the Arp2/3 complex exert outward force on the plasma membrane, allowing B cells to form membrane protrusions that can scan the surface of antigen-presenting cells (APCs). The resulting Arp2/3 complex-dependent actin retrograde flow promotes the centripetal movement and progressive coalescence of BCR microclusters, which amplifies BCR signaling. Glia maturation factor γ (GMFγ) is an actin disassembly-protein that releases Arp2/3 complex-nucleated actin filaments from actin networks. By doing so, GMFγ could either oppose the actions of the Arp2/3 complex or support Arp2/3 complex-nucleated actin polymerization by contributing to the recycling of actin monomers and Arp2/3 complexes. We now show that reducing the levels of GMFγ in human B cell lines via transfection with a specific siRNA impairs the ability of B cells to spread on antigen-coated surfaces, decreases the velocity of actin retrograde flow, diminishes the coalescence of BCR microclusters into a central cluster at the B cell-APC contact site, and decreases APC-induced BCR signaling. These effects of depleting GMFγ are similar to what occurs when the Arp2/3 complex is inhibited. This suggests that GMFγ cooperates with the Arp2/3 complex to support BCR-induced actin remodeling and amplify BCR signaling at the immune synapse.

## Introduction

Activated B cells contribute to health and disease by producing antibodies, secreting pro- and anti-inflammatory cytokines, and presenting antigens (Ags) to T cells (Conley et al., 2009; Shen and Fillatreau, 2015; Cashman et al., 2019; Cyster and Allen, 2019; Meffre and O’Connor, 2019). B cell activation is initiated by the binding of Ags to the B cell Ag receptor (BCR), which triggers signaling pathways that regulate gene expression, cell metabolism, cell cycle progression, and cytoskeletal organization (Packard and Cambier, 2013; Li et al., 2018; Jellusova, 2020). BCR signaling induces reorganization of the actin cytoskeleton, which can be readily visualized *in vitro* when B cells are plated on a rigid substrate coated with Ags or with antibodies against the membrane immunoglobulin (Ig) subunit of the BCR. Under these conditions, B cells spread in a radial manner, forming a peripheral ring of branched filamentous actin (F-actin) that generates broad, outwardly moving lamellipodial protrusions (Freeman et al., 2011). At the same time, the central region of the Ag contact site is depleted of F-actin via the action of actin-disassembly proteins such as cofilin (Freeman et al., 2011). Many actin-regulatory proteins are targets of BCR signaling (Tolar, 2017) and mutations in actin regulators such as Wiskott-Aldrich Syndrome protein (WASp), Arpc1B, Hem1/NCKAP1L, and Wdr1 result in autoimmune or immunodeficiency syndromes that have been termed actinopathies (Kile et al., 2007; Kahr et al., 2017; Kuijpers et al., 2017; Brigida et al., 2018; Candotti, 2018; Pfajfer et al., 2018; Randzavola et al., 2019; Volpi et al., 2019; Cook et al., 2020; Sprenkeler et al., 2020). Hence, identifying proteins that link the BCR to actin remodeling can provide new insights into B cell activation and dysfunction.

Although B cells can be activated by soluble Ags, they are activated most efficiently by Ags that are displayed on the surface of Ag-presenting cells (APCs) (Batista and Harwood, 2009; Cyster, 2010; Heesters et al., 2016). Follicular dendritic cells, dendritic cells, and subcapsular sinus macrophages can capture Ags and concentrate them on their surface in an intact form that can be recognized by B cells (Heesters et al., 2016). When B cells bind Ags that are mobile within a membrane, BCR signaling stimulates rapid remodeling of the actin cytoskeleton, as well as actin-dependent spatial reorganization of BCRs and other membrane proteins, leading to formation of an immune synapse (Harwood and Batista, 2011; Song et al., 2014).

The actin remodeling that drives immune synapse formation enhances the ability of membrane-bound Ags to stimulate BCR signaling and B cell activation (Depoil et al., 2008; Bolger-Munro et al., 2019). Initial BCR signaling initiates transient, localized disassembly of the submembrane actin mesh (Freeman et al., 2011). This removes actin-based diffusion barriers and the resulting increase in BCR mobility within the plasma membrane enables them to form BCR microclusters (Treanor et al., 2010, 2011; Freeman et al., 2011). BCR clustering leads to phosphorylation of the immunoreceptor tyrosine-based activation motifs (ITAMs) within the CD79a/b (Igα/Igβ) subunit of the BCR (Dal Porto et al., 2004; Abraham et al., 2016). Subsequent recruitment of the Syk tyrosine kinase and other signaling proteins to the BCR leads to the formation of microcluster-based signaling complexes termed microsignalosomes (Weber et al., 2008; Treanor et al., 2009). Concomitantly, actin polymerization at the cell periphery allows B cells to extend membrane protrusions across the surface of the APC in order to encounter more Ag and form additional BCR microclusters (Fleire et al., 2006; Bolger-Munro et al., 2019). The B cell then retracts these membrane protrusions, promoting the centripetal movement and coalescence of BCR microclusters (Fleire et al., 2006; Bolger-Munro et al., 2019). BCR-Ag microclusters ultimately coalesce into a central supramolecular activation complex (cSMAC), a distinguishing feature of an immune synapse. cSMAC formation may facilitate the internalization of BCR-Ag complexes, which allows B cells to present Ags to T cells and elicit critical second signals for activation (Yuseff et al., 2013; Nowosad et al., 2016).

There are two major modes of actin network assembly (Kadzik et al., 2020). Formin proteins mediate linear actin polymerization, which generates thin membrane protrusions such as filopodia. In contrast, the assembly of branched actin networks, which is initiated by the actin-related protein (Arp) 2/3 complex, drives the formation of broad lamellipodial protrusions. When activated by WASp or other nucleation-promoting factors, the Arp2/3 complex binds to the side of an actin filament and nucleates the formation of a new actin branch that grows at a 70° angle from the mother filament.

We have shown that the Arp2/3 complex plays a major role in BCR-induced actin remodeling, immune synapse formation, and APC-induced BCR signaling (Bolger-Munro et al., 2019). When the Arp2/3 complex is inhibited or depleted, B cells exhibit impaired spreading on immobilized anti-Ig antibodies. We also showed that Arp2/3 complex-dependent actin retrograde flow, a consequence of membrane-proximal actin polymerization being opposed by the elastic resistance of the plasma membrane, is required for the initial centripetal movement of BCR microclusters and for cSMAC formation. Importantly, this Arp2/3 complex-dependent movement and coalescence of BCR microclusters amplifies BCR signaling and promotes B cell activation. These findings suggest that other proteins that regulate Arp2/3 complex-nucleated actin polymerization are also likely to be important for B cell spreading and APC-induced B cell responses.

The remodeling of actin networks involves the cooperative actions of actin assembly and disassembly factors. In lamellipodia, Arp2/3 complex-dependent actin polymerization depends on actin-disassembly factors such as cofilin, coronins, and glia maturation factor γ (GMFγ) (Goode et al., 2018; Kadzik et al., 2020). These proteins disassemble older segments of actin filaments, releasing actin monomers that can then be loaded with ATP and used for new actin polymerization. This coupling of actin depolymerization and polymerization is referred to as treadmilling (Carlier and Shekhar, 2017).

The role of GMFγ in B cells, and in immune synapse formation, has not been investigated. In *S. pombe*, disrupting *Gmf1*, the gene encoding the homolog of GMFγ, results in reduced actin turnover and defects in actin organization (Ydenberg et al., 2015). GMFγ also regulates actin dynamics in immune cells. GMFγ depletion reduces the migration of neutrophils, T cells, and monocytes toward chemoattractants (Aerbajinai et al., 2011, 2016; Lippert and Wilkins, 2012). In *in vitro* assays, GMFγ can inhibit Arp2/3 complex activity and also debranch actin networks by causing the release of the Arp2/3 complex and daughter filament from the mother filament (Gandhi et al., 2010; Nakano et al., 2010; Ydenberg et al., 2013; Sokolova et al., 2017). These activities could position GMFγ as an inhibitor of processes that depend on Arp2/3 complex-nucleated actin polymerization. Alternatively, GMFγ-mediated release of Arp2/3 complex-bound filaments from the actin network could sustain Arp2/3 complex-dependent processes by enabling the recycling of both Arp2/3 complexes and actin monomers (Goode et al., 2018). Given its potential role as either a positive or negative regulator of Arp2/3 complex-dependent actin polymerization, we tested the hypothesis that GMFγ regulates BCR-induced actin remodeling, immune synapse formation, and APC-induced BCR signaling.

## Materials and Methods

### B Cell Lines

The Ramos human IgM^+^ B cell line was obtained from ATCC (#CRL-1596). Raji D1.3 B cells, which express a transgenic hen egg lysozyme (HEL)-specific BCR were a gift from Dr. Bebhinn Treanor (University of Toronto, Toronto, Canada). A population of Raji D1.3 B cells with high expression of the D1.3 IgM-BCR was obtained by FACS sorting the cells after staining with rat anti-mouse IgM-FITC (Invitrogen, #11-5790-81, 1:200 dilution). Cells were cultured in RPMI-1640 supplemented with 10% heat-inactivated fetal calf serum (FCS), 1 mM sodium pyruvate, 2 mM glutamine, and 50 μM β-mercaptoethanol. All cells were confirmed to be mycoplasma-negative.

### siRNA-Mediated Depletion of GMFγ

Using the Ingenio electroporation kit (Mirus, #MIR50118) and an Amaxa Nucleofector (program O-006 for Ramos B cells; program M-013 for Raji D1.3 B cells), 3 × 10^6^ cells were transiently transfected with 2 μg of either control non-targeting siRNA (Silencer Select Negative Control #2, Ambion, #4390846) or siRNA directed against human GMFγ (GMFG Silencer Select Pre-designed siRNA, Ambion, #4392420). Where indicated, the cells were co-transfected with 1 μg of the pmaxGFP plasmid (Lonza, #D-00069). Transfected cells were cultured for 48–72 h before being used in experiments. The levels of GMFγ and actin (loading control) in siRNA-transfected cells were analyzed by immunoblotting with a rabbit antibody against human GMFγ (Proteintech, #13625-1-AP, 1:500; overnight at 4°C) or a monoclonal β-actin antibody (Santa Cruz, #sc-47778, 1:5000; 1 h at room temperature), followed by horseradish peroxidase-conjugated goat anti-rabbit IgG (Bio-Rad, #170-6515; 1:3,000) or goat anti-mouse IgG (Bio-Rad, #170-6516; 1:3,000). Bands were detected by ECL (Azure Biosystems, #AC2101), then imaged and quantified using a Li-Cor C-DiGit imaging system.

### Analysis of Cell Surface BCR Levels and Cell Size by Flow Cytometry

To assess cell surface BCR levels, 10^6^ B cells were resuspended in 50 μL ice-cold FACS buffer (PBS with 2% FCS), and then stained on ice for 30 min with Alexa Fluor 647-conjugated goat anti-human IgM Fab fragments (Jackson ImmunoResearch, #109-607-043, 1:200) or with rat anti-mouse IgM FITC (Invitrogen, #11-5790-81, 1:200) to detect the D1.3 BCR. Flow cytometry was performed using an LSRII-561 cytometer (Becton Dickinson Biosciences). Data were analyzed using FlowJo software (Treestar Inc.) using forward and side scatter to gate on single intact cells. Forward scatter was used as a relative measure of cell size.

### Analysis of B Cell Spreading, GMFγ Localization, and Actin Dynamics

Glass coverslips (12-mm diameter, Thermo Fisher Scientific, #12-545-80) were coated with either 2.5 μg/cm^2^ donkey anti-human IgM (Jackson ImmunoResearch, #709-005-073) or 0.22 μg/cm^2^ HEL (NANOCS, #LSN-BN-1) for 30 min at room temperature and then blocked with 2% bovine serum albumin (BSA) in PBS for 30 min at room temperature. After being resuspended in modified HEPES-buffered saline (mHBS; 25 mM HEPES, pH 7.2, 125 mM NaCl, 5 mM KCl, 1 mM CaCl_2_, 1 mM Na_2_HPO_4_, 1 mg/mL glucose, 1 mM sodium pyruvate, 2 mM glutamine, 50 μM β-mercaptoethanol) with 2% FCS, 5 × 10^4^ B cells were added to the coverslips. After 3–30 min at 37°C, the cells were fixed with 4% paraformaldehyde (PFA) for 10 min at room temperature and then permeabilized with 0.1% Triton X-100 in PBS for 3 min. F-actin was visualized by staining with rhodamine-phalloidin (Thermo Fisher, #R415, 1:400 in PBS + 2% BSA) for 30 min at room temperature. Where indicated, the fixed cells were blocked with PBS + 2% BSA for 30 min, stained for 1 h at room temperature with rabbit anti-human GMFγ (Proteintech, #13625-1-AP, 1:200 in PBS + 2% BSA), washed, and then incubated for 30 min at room temperature with Alexa Fluor-647-conjugated goat anti-rabbit IgG (Thermo Fisher Scientific, #A21244, 1:400 in PBS + 2% BSA) plus rhodamine-phalloidin. Coverslips were mounted onto slides using ProLong Diamond anti-fade reagent (Thermo Fisher Scientific, #P36965). Images of the B cell-coverslip interface were captured using a spinning disk confocal microscope (Intelligent Imaging Innovations) consisting of an inverted Zeiss Axiovert 200M microscope with a 100× 1.45 NA oil Plan-Fluor objective lens and a QuantEM 512SC Photometrics camera. Cell area was quantified using Fiji software (Schindelin et al., 2012). Radial fluorescence intensity profiles were generated with a custom macro that utilizes Fiji software together with the Radial Profile Extended plug-in^1^. This plug-in is based on an algorithm originally developed by Baggethun^2^, which yields a plot of the normalized integrated intensities around concentric circles, as a function of the distance from a center point. The integrated intensity is the sum of the fluorescence intensity values for the pixels around a circle of radius *x*, divided by the number of these pixels that are part of the image (i.e., not outside the cell). This value is then normalized to the maximal integrated intensity for any circle from that cell. The radial coordinates for each cell are expressed as normalized distances from the center.

For live-cell imaging at 37°C, Raji D1.3 B cells were transfected with a plasmid encoding F-tractin-GFP (Johnson and Schell, 2009), along with either control siRNA or GMFγ siRNA, and then cultured for 72 h. Cells (2 × 10^5^ in 100 μL mHBS + 2% FCS) were added to coverslips that had been coated with 2.5 μg/cm^2^ donkey anti-human IgM and allowed to spread for 5 min. The cell-coverslip contact site was then imaged by total internal reflection fluorescence (TIRF) microscopy. Images of GFP-expressing cells were acquired every 2 s for 15 min using an Olympus IX81 inverted microscope equipped with a 150× NA 1.45 TIRF objective, a high performance electron multiplier charge-coupled device camera (Photometrics Evolve), and real-time data acquisition software (Metamorph). Fiji software was used to generate kymographs.

### APC-Induced cSMAC Formation and BCR Signaling

B cell-APC interactions were analyzed as described previously (Wang et al., 2018; Bolger-Munro et al., 2019). COS-7 cells (ATCC, #CRL-1651) were transiently transfected with a plasmid encoding the mHEL-HaloTag Ag. The mHEL-HaloTag protein consists of the complete HEL protein fused to the transmembrane and cytosolic domains of the H-2K^b^ protein, with the HaloTag protein fused to the C-terminus of the H-2K^b^ cytosolic domain (Wang et al., 2018). The mHEL-HaloTag-expressing COS-7 cells were cultured for 24 h before adding 2.2 × 10^4^ cells to glass coverslips that had been coated with 5 μg/mL fibronectin, and then culturing the cells for an additional 24 h. After washing the COS-7 cells with PBS, the mHEL-HaloTag Ag was labeled with Janelia Fluor 549 HaloTag ligand (Promega, #GA1110, 1:20,000 dilution in 200 μL mHBS + 2% FCS) for 15 min at 37°C. siRNA-transfected Raji D1.3 cells (5 × 10^5^ cells in 100 μL mHBS + 2% FCS) were added to the COS-7 APCs for 3–30 min at 37°C. The cells were then fixed with 4% PFA for 10 min, permeabilized with 0.1% Triton X-100 in PBS for 3 min, and blocked with PBS + 2% BSA for 30 min, all at room temperature. The cells were stained for 1 h at room temperature with an antibody that recognizes the phosphorylated CD79 ITAMs (pCD79; Cell Signaling Technologies, #5173, 1:200 in PBS + 2% BSA), washed, and then incubated for 30 min at room temperature with PBS + 2% BSA containing Alexa Fluor-647-conjugated goat anti-rabbit IgG (Thermo Fisher Scientific, #A21244, 1:400) plus Alexa Fluor 488-conjugated phalloidin (Thermo Fisher Scientific, #A12379, 1:400). After mounting the coverslips onto slides, the B cell-APC interface was imaged by spinning disk confocal microscopy. For each B cell, custom Fiji macros^3^ were used to quantify the total amount of pCD79 fluorescence and mHEL-HaloTag Ag fluorescence present in clusters at the B cell-APC interface, as well as the Ag fluorescence intensity for each microcluster on an individual B cell (Bolger-Munro et al., 2019, 2021). Briefly, the mean background fluorescence intensity per pixel was calculated and subtracted from all pixel values by using the rolling ball background subtraction ImageJ plug-in with the radius of the rolling ball set to 10 pixels. A binary image was then generated using Otsu thresholding. Cluster segmentation was performed using the “Analyze Particles” function in FIJI with clusters defined as being > 0.05 μm^2^. Masks generated in this way were then mapped onto the original image for quantification of pixel intensity. Pixel intensities within the mask were summed to determine the total fluorescence intensity that was present in clusters for each cell. As defined previously, a cell was deemed to have formed a cSMAC when > 90% of the Ag fluorescence had been gathered into one or two clusters at the center of the synapse (Bolger-Munro et al., 2019).

### BCR Signaling in Response to Soluble Anti-IgM

Raji D1.3 B cells (3 × 10^6^ in 100 μL mHBS) were stimulated at 37°C with 20 μg/mL goat anti-mouse IgM (Jackson ImmunoResearch, #115-005-020) to engage the D1.3 BCR. Ramos B cells were stimulated with 20 μg/mL donkey anti-human IgM (Jackson ImmunoResearch, #709-005-073). Reactions were stopped, and cells lysed, by adding 30 μL RIPA buffer (30 mM Tris-HCl, pH 7.4, 150 mM NaCl, 1% Igepal (Sigma-Aldrich), 0.5% sodium deoxycholate, 0.1% SDS, 2 mM EDTA) with 3X protease and phosphatase inhibitors (3 mM phenylmethylsulfonyl fluoride, 30 μg/mL leupeptin, 3 μg/mL aprotinin, 3 μg/mL pepstatin A, 75 mM β-glycerophosphate, 3 mM Na_3_MoO_4_, 3 mM Na_3_VO_4_). After 15 min on ice with intermittent mixing, insoluble material was removed by centrifugation. Cell lysates (20 μg protein) were separated by SDS-PAGE and then analyzed by immunoblotting with antibodies that recognize the phosphorylated CD79 ITAMs (pCD79; Cell Signaling Technologies, #5173, 1:000; overnight at 4°C), human GMFγ (Proteintech, #13625-1-AP, 1:500; overnight at 4°C) or β-actin (Santa Cruz, #sc-47778, 1:5,000; 1 h at room temperature), followed by horseradish peroxidase-conjugated goat anti-rabbit IgG (Bio-Rad, #170-6515; 1:3,000) or goat anti-mouse IgG (Bio-Rad, #170-6516; 1:3,000). After ECL detection, blots were quantified and imaged using a Li-Cor C-DiGit imaging system.

### Statistical Analysis

Two-tailed paired *t*-tests were used to compare mean values for matched sets of samples. The Mann-Whitney *U*-test was used to compare ranked values in samples with many cells and high variability (e.g., dot plots for immunofluorescence signaling data). Outliers were identified using Robust Regression and Outlier Removal (ROUT) in GraphPad Prism with Q set to 1% (Motulsky and Brown, 2006).

## Results

### Depleting GMFγ Reduces B Cell Spreading on Immobilized Anti-IgM or Ag

**FIGURE 1:**
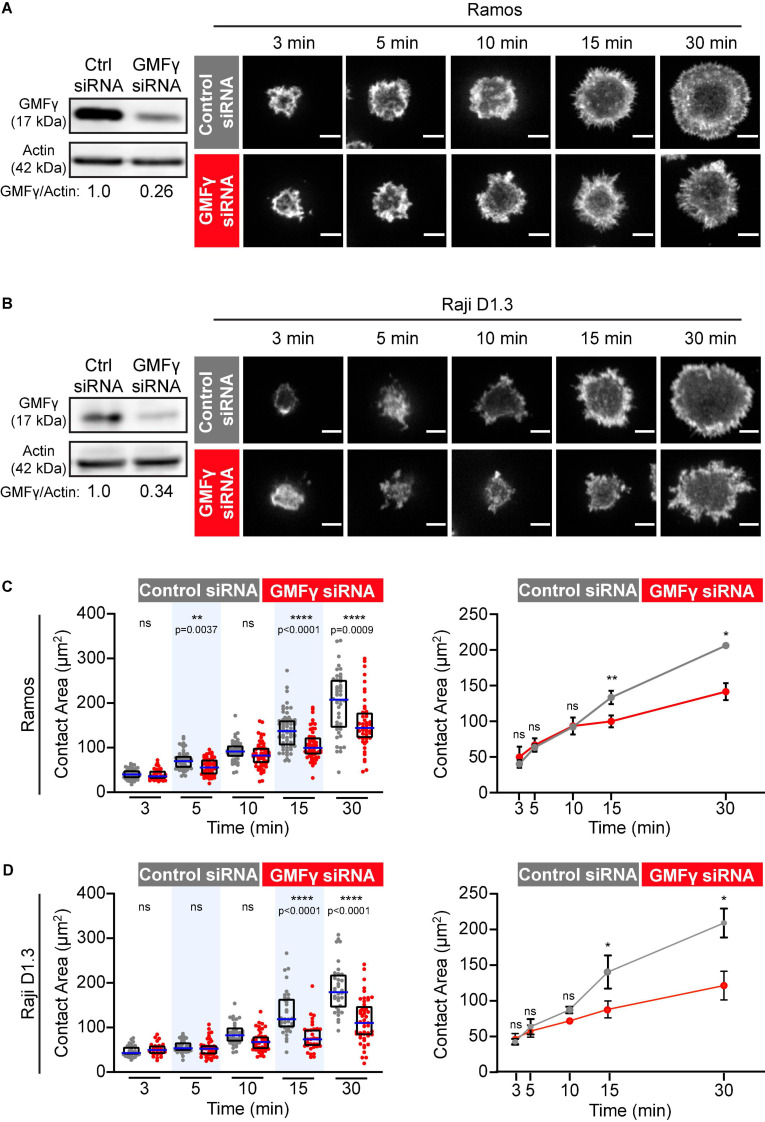
Depleting GMFγ reduces B cell spreading on immobilized anti-Ig. **(A,B)** Ramos B cells **(A)** or Raji D1.3 B cells **(B)** were transfected with control non-targeting siRNA or GMFγ siRNA. Immunoblots (left panels) show GMFγ levels in cell extracts, with actin as a loading control. The transfected B cells were added to anti-IgM-coated-coverslips and allowed to spread for the indicated times before being stained with rhodamine-phalloidin. Representative confocal microscopy images are shown (right panels). Scale bars: 5 μm. **(C,D)** For each B cell, the cell area was quantified using the actin staining to define the cell edge. The left panels show data from representative experiments. Each dot represents one cell. The median (blue line) and interquartile ranges (black box) for > 30 cells are shown for each time point. *p*-values were determined using the Mann-Whitney *U*-test. The right panels show the mean ± SEM of the median cell areas from three independent experiments. Where no error bars are shown, they were smaller than the symbols. *p*-values were determined using paired two-tailed *t*-tests. ****p < 0.0001; **p < 0.01; *p ≤ 0.05; ns, not significant (*p* > 0.05).

To assess the role of GMFγ in B cells, we transfected the Ramos and Raji D1.3 human B cell lines with either GMFγ-specific siRNA or a control non-targeting siRNA. Representative GMFγ immunoblots are shown in Figures 1A,B. Quantification of immunoblots from 17 independent experiments showed that transfection with GMFγ siRNA reduced the level of GMFγ protein to 25–50% of that in control siRNA-transfected cells. This partial depletion of GMFγ could result in underestimating the contributions of GMFγ to B cell responses. Partial depletion of GMFγ has been reported in a number of other studies that have employed GMFγ siRNA as a loss-of-function approach (Lippert and Wilkins, 2012; Wang et al., 2014; Aerbajinai et al., 2016, 2019).

GMFγ has been implicated in the recycling of receptors back to the cell surface after endocytosis (Lippert and Wilkins, 2012; Aerbajinai et al., 2013, 2016) and depleting GMFγ in macrophages increases cell surface levels of Toll-like receptor 4 (Aerbajinai et al., 2013). Therefore, prior to assessing the role of GMFγ in BCR-induced actin remodeling, we tested whether GMFγ depletion altered BCR surface expression. Cell surface BCR levels in GMFγ siRNA-transfected Ramos B cells were 95 ± 6% of those in control siRNA-transfected cells (mean ± SEM for the mean fluorescence intensities from 5 experiments) (Supplementary Figure 1A). In Raji D1.3 B cells, GMFγ siRNA transfection reduced cell surface levels of the endogenous human BCR to 79 ± 4% of those in control siRNA-transfected cells (mean ± SEM for 5 experiments) and the transfected D1.3 BCR to 76 ± 2% of control cell levels (mean ± SEM for 4 experiments) (Supplementary Figure 1B). The reduced BCR cell surface expression in Raji D1.3 cells was not due to a decrease in cell size as control and GMFγ-depleted cells had similar mean forward scatter values (Supplementary Figure 1). Importantly, compared to control cells, GMFγ siRNA-transfected Ramos and Raji D1.3 B cells did not exhibit a reduction in initial BCR signaling (CD79 phosphorylation) in response to soluble anti-Ig antibodies. This finding is discussed in more detail in the last paragraph of the results section.

When B cells encounter anti-Ig antibodies or Ags that have been immobilized on rigid surfaces, BCR signaling stimulates actin remodeling that drives radial B cell spreading. This spreading is substantially reduced or altered when either cofilin or the Arp2/3 complex is inhibited (Freeman et al., 2011; Bolger-Munro et al., 2019, 2021). Hence, this assay is a robust platform for identifying proteins that regulate peripheral actin dynamics in B cells. To test whether GMFγ regulates BCR-induced actin remodeling and B cell spreading, control siRNA- and GMFγ siRNA-transfected B cells were added to anti-IgM-coated coverslips and allowed to spread for 3–30 min. Because the incomplete knockdown of GMFγ could be due to some cells not taking up the siRNA, we co-transfected the B cells with a GFP-encoding plasmid and then analyzed only GFP-expressing cells. The cell periphery was visualized by staining F-actin and the cell area in the confocal plane closest to the coverslip was quantified using Fiji software. Ramos and Raji D1.3 B cells that had been transfected with GMFγ siRNA exhibited relatively normal initial cell spreading at 3–10 min (Figure 1). However, compared to control siRNA-transfected cells, GMFγ-depleted Ramos and Raji D1.3 B cells had significantly reduced contact areas at the 15 and 30 min time points (Figure 1). These findings suggest that GMFγ is a positive regulator of sustained B cell spreading.

**FIGURE 2:**
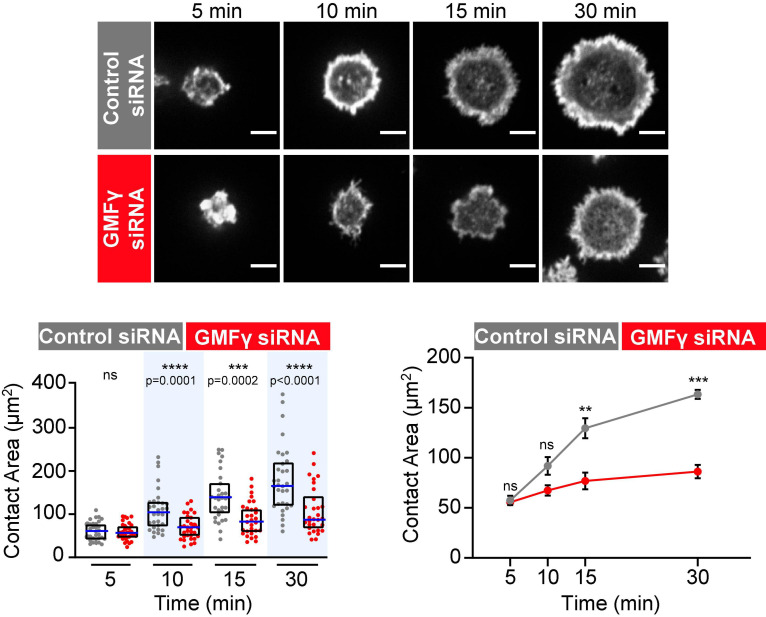
Depleting GMFγ reduces B cell spreading on immobilized HEL. Raji D1.3 B cells that had been transfected with either control siRNA or GMFγ siRNA were added to coverslips coated with 0.22 μg/cm^2^ HEL and allowed to spread for the indicated times. Representative confocal microscopy images of cells stained with rhodamine-phalloidin are shown. Scale bars: 5 μm. The cell area was quantified using the actin staining to define the cell edge. In the dot plot (left graph), each dot represents one cell, the median (blue line) and interquartile ranges (black box) for > 30 cells are shown for each time point, and *p*-values were determined using the Mann-Whitney *U*-test. The line graph on the right shows the mean ± SEM of the median cell areas from three independent experiments. Where no error bars are shown, they were smaller than the symbols. *p*-values were determined using paired two-tailed *t*-tests. ****p < 0.0001; ***p < 0.001; **p < 0.01; ns, not significant (*p* > 0.05).

Because the Raji D1.3 B cells express a transfected HEL-specific BCR in addition to their endogenous BCR, we were able to extend these studies to Ag-induced B cell spreading. Recent work has shown that occupancy of the Ag-binding sites in the BCR may activate the BCR in a different manner than anti-Ig antibodies that cluster BCRs (Volkmann et al., 2016; Shen et al., 2019). We found that depleting GMFγ impaired the ability of Raji D1.3 B cells to spread on HEL-coated coverslips, with significant and consistent reductions in the median spreading areas at the 15 and 30 min time points compared to control siRNA-transfected cells (Figure 2). Thus, GMFγ is important for sustained B cell spreading on both anti-Ig and Ag.

### GMFγ Abuts the Inner Face of the Peripheral Actin Ring and Contributes to Actin Retrograde Flow

**FIGURE 3:**
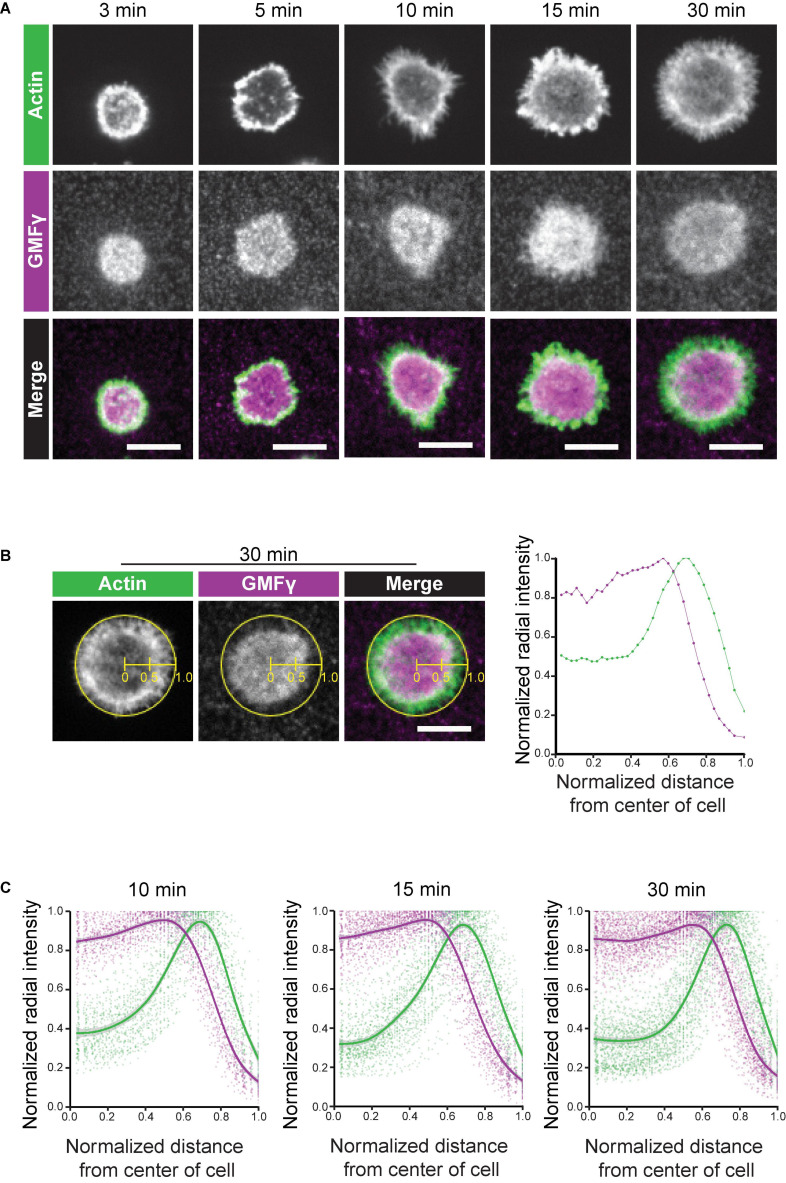
GMFγ abuts the inner face of the peripheral actin ring. Ramos B cells were allowed to spread for 3–30 min on anti-IgM-coated coverslips before being stained with an anti-GMFγ antibody plus rhodamine-phalloidin. **(A)** Representative confocal microscopy images. Scale bars: 10 μm. **(B)** Example of actin and GMFγ radial fluorescence intensity profiles for an individual cell. The ImageJ Radial Profile Extended plug-in generates concentric circles with radii corresponding to different distances from the center of the cell and then calculates the average fluorescence intensity per pixel around the perimeter of each circle. This integrated radial intensity value is normalized to the maximal value for any circle from that cell (defined as 1.0) and is plotted vs. its relative distance between the center of the cell (*x* = 0) and the edge of the cell (*x* = 1). The graph (right panel) shows the radial intensity profiles for GMFγ (purple) and F-actin (green) for the cell shown in the left three panels, which had spread on immobilized anti-IgM for 30 min. **(C)** The averaged radial fluorescence intensity profiles for 78–129 cells per time point are shown for cells that had spread on anti-IgM for 10, 15, or 30 min in one of 4 independent experiments. For each relative distance, the dots are the values for individual cells. The solid lines and corresponding gray contours are the generalized additive model and standard error of the smoothing estimate.

The radial spreading of B cells in response to immobilized BCR ligands is characterized by the formation of a peripheral branched actin network that drives lamellipodial protrusion. Persistent outward movement of lamellipodia is associated with actin treadmilling in which actin-disassembly factors dismantle older segments of the peripheral actin network that are further from the plasma membrane, allowing components to be recycled for new actin assembly at the plasma membrane (Carlier and Shekhar, 2017). *In vitro*, GMFγ preferentially releases older actin branches where the Arp2/3-bound ATP has been hydrolyzed to ADP (Boczkowska et al., 2013; Pandit et al., 2020). To gain insight into how GMFγ regulates peripheral actin dynamics during B cell spreading we imaged the localization of the endogenous GMFγ protein, relative to actin structures, in Ramos B cells that were plated on immobilized anti-Ig (Figure 3). As the B cells spread, they formed a distinct peripheral F-actin ring surrounding a central actin-depleted region. GMFγ was present in this central region of the cell and abutted the interior face of the peripheral F-actin ring (Figures 3A,B). In contrast, GMFγ appeared to be relatively under-represented in the outer portion of the peripheral actin ring, which is presumably close to the plasma membrane.

To quantitatively assess the spatial distribution of GMFγ relative to the peripheral actin ring, we generated radial fluorescence intensity profiles in which the normalized integrated fluorescence intensity is plotted vs. the relative distance from the center of the cell (x = 0) to the edge of the cell (x = 1.0). Briefly, the algorithm generates a series of concentric circles around the center of the cell and then determines the average fluorescence intensity for the pixels along the perimeter of each circle. This integrated intensity is then normalized to the maximal integrated intensity (defined as 1.0) for any circle from that cell, and plotted vs. the relative distance from the center of the cell. Figure 3B shows a representative example for an individual cell that had spread on immobilized anti-IgM for 30 min. In Figure 3C, Ramos B cells were allowed to spread on immobilized anti-IgM for 10, 15, or 30 min and the averaged radial fluorescence intensity profiles for 78–129 cells per time point are shown. The peripheral actin ring that forms in spreading B cells coincided with a distinct peak of the actin radial fluorescence intensity, which was close to the edge of the cell (Figures 3B,C). Importantly, this analysis showed that the peak of GMFγ fluorescence intensity was consistently closer to the center of the cell than the peak of actin fluorescence (Figures 3B,C). Although GMFγ was abundant in the center of the cells, GMFγ levels were substantially lower where the actin fluorescence peaked. These findings are consistent with a model in which GMFγ disassembles older portions of the peripheral actin network that are closer to the center of the cell. At the same time, the reduced levels of GMFγ at the leading edge would allow Arp2/3 complex-nucleated branched actin polymerization to drive outward expansion of lamellipodia.

**FIGURE 4:**
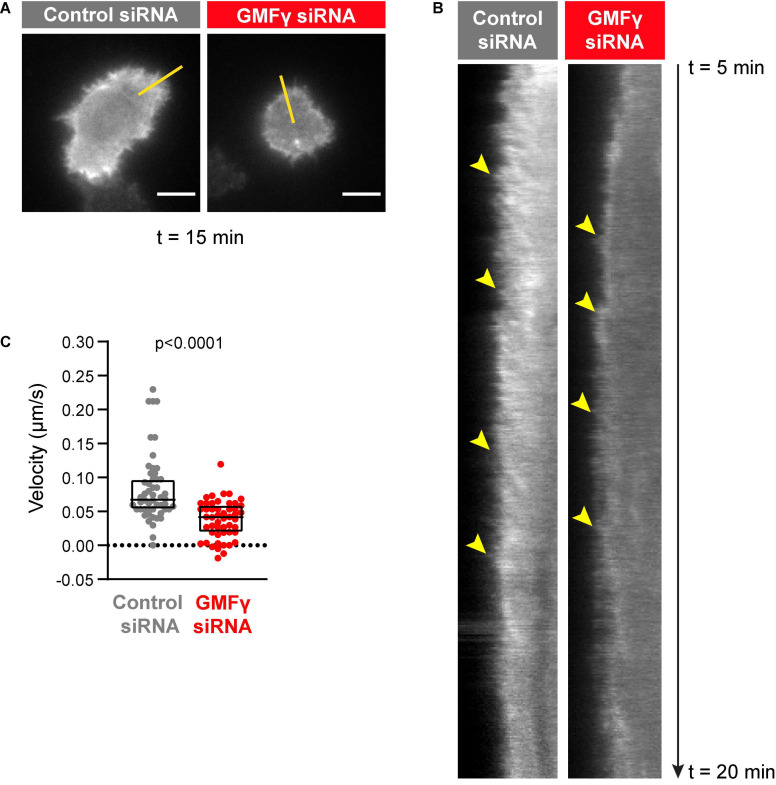
Depleting GMFγ reduces the velocity of actin retrograde flow. Raji D.3 B cells that had been co-transfected with F-tractin-GFP cDNA and either control siRNA or GMFγ siRNA were added to anti-human IgM-coated coverslips and allowed to spread for 5 min. The cells were then imaged by TIRF microscopy at 2 s intervals for 15 min (*t* = 5 min to *t* = 20 min). Video recordings of representative cells are shown in Supplementary Movie 1 (control siRNA) and Supplementary Movie 2 (GMFγ siRNA). **(A)** Still images of the control siRNA- and GMFγ siRNA-transfected cells shown in Supplementary Movies 1, 2 at *t* = 15 min. Scale bars: 5 μm. **(B)** Kymographs were generated along the yellow lines in **(A)**. Arrowheads indicate the starting points of representative actin tracks for which centripetal velocities were determined. **(C)** The centripetal velocity (Δx/Δt) was calculated for individual actin tracks on the kymographs. Each dot on the graph is an individual actin track. The velocity was determined for 52 tracks from 9 cells (control siRNA) or 51 tracks from 11 cells (GMFγ siRNA). The Mann-Whitney *U*-test was used to calculate the *p*-value.

The outward forces generated by actin polymerization at the plasma membrane are opposed by the elastic resistance of the membrane. The resulting inward forces result in retrograde flow of the peripheral actin network. When B cells interact with Ags that are mobile within a membrane, this actin retrograde flow promotes the initial centralization and progressive coalescence of BCR microclusters, which amplifies microcluster-based BCR signaling (Bolger-Munro et al., 2019). Because actin disassembly supports on-going actin polymerization at the plasma membrane, we asked whether GMFγ contributes to the generation of actin retrograde flow in B cells. To assess this, real-time imaging of peripheral F-actin structures was carried out in Raji D1.3 B cells that were transfected with control siRNA or GMFγ siRNA along with cDNA encoding F-tractin-GFP, a fusion protein that binds F-actin. This allowed us to visualize the centripetal movement of peripheral actin structures (Figure 4A and Supplementary Movies 1, 2). We then used kymograph analysis to calculate the inward velocity of distinct actin structures from multiple cells (Figure 4B). This analysis showed that the median velocity of the actin retrograde flow was reduced by 38% when the cells were transfected with GMFγ siRNA, as compared to control siRNA-transfected cells (Figure 4C). Thus, GMFγ contributes to the peripheral actin dynamics that result in actin retrograde flow.

### Depleting GMFγ Reduces APC-Induced cSMAC Formation and BCR Signaling at the Immune Synapse

When B cells encounter APCs displaying Ags that can bind to their BCR, BCR-Ag microclusters form rapidly and begin to coalesce. Arp2/3 complex-dependent actin retrograde flow is required for the initial centripetal movement of BCR-Ag microclusters, which amplifies microcluster-based BCR signaling and promotes the microcluster coalescence that leads to cSMAC formation (Bolger-Munro et al., 2019). Because GMFγ supports the Arp2/3 complex-dependent spreading of B cells on immobilized BCR ligands, we asked whether it is also important for APC-induced immune synapse formation and BCR signaling. To test this, HEL-specific Raji D1.3 B cells were transfected with either control or GMFγ siRNA and then allowed to interact with adherent APCs expressing the mHEL-HaloTag Ag, a transmembrane form of HEL that can be fluorescently labeled (Wang et al., 2018; Bolger-Munro et al., 2019). After 3–30 min, the cells were fixed and stained with an antibody to the phosphorylated CD79a/CD79b ITAMs in order to detect this essential initial event in BCR signaling. Imaging the B cell-APC interface allowed us to visualize BCR-Ag microclusters, monitor their coalescence into a cSMAC, and quantify the amount of pCD79 and mHEL-HaloTag fluorescence that is present in clusters. As shown in previous studies, the gathering of membrane-bound Ags into microclusters that can be detected by diffraction-limited microscopy is due to BCR binding. These Ag clusters align with BCR clusters on the B cell, and clusters of activated BCRs (i.e., those in which CD79 is phosphorylated or to which the Syk protein kinase has been recruited) overlap extensively with the Ag clusters (Depoil et al., 2008). Hence, Ag clusters that align with pCD79 clusters are believed to be clusters of BCR-bound Ags in which Ag binding has initiated BCR signaling.

**FIGURE 5:**
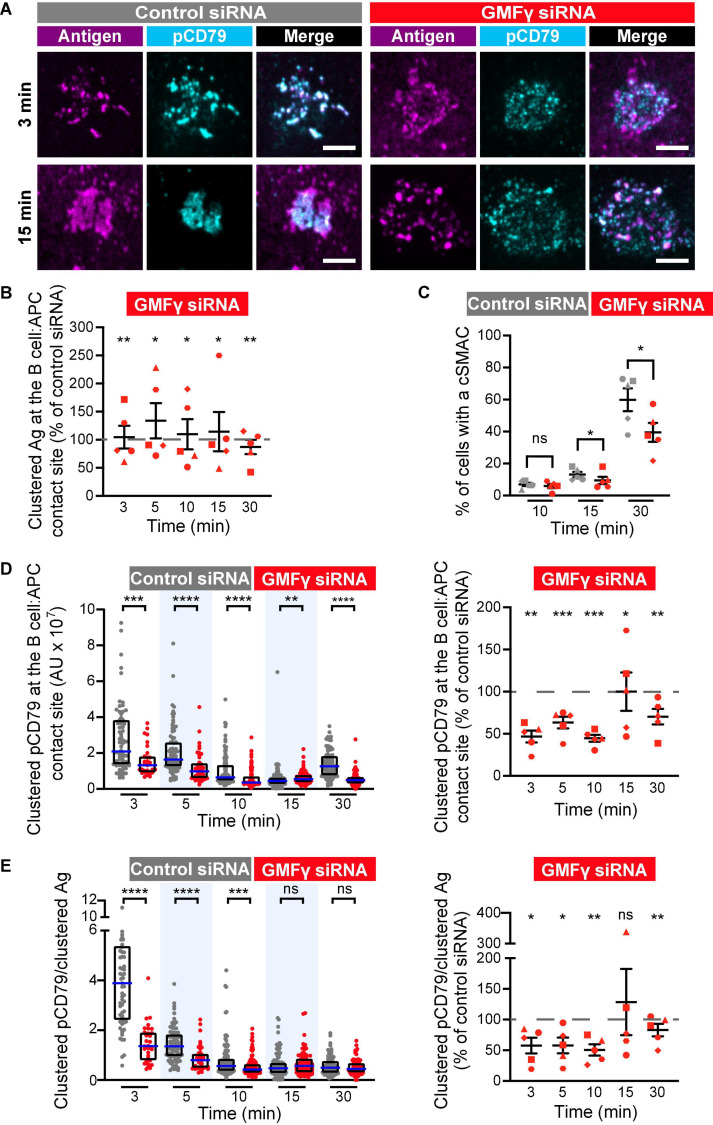
Depleting GMFγ reduces cSMAC formation and proximal BCR signaling at the immune synapse. Raji D1.3 B cells were transfected with either control siRNA or GMFγ siRNA and added to COS-7 APCs expressing the mHEL-HaloTag Ag (magenta). The cells were fixed at the indicated times and stained with an antibody that recognizes the phosphorylated CD79 ITAMs (pCD79, cyan). The B cell-APC interface was imaged by spinning disk microscopy. **(A)** Representative images from one of five independent experiments. Scale bars: 5 μm. **(B)** The total fluorescence intensity of the mHEL-HaloTag Ag that had been gathered into clusters at the B cell-APC contact site was quantified for each B cell and the median values were calculated for each time point. Each symbol on the graph represents the median value for the GMFγ knockdown cells, expressed as a percent of the median value for the control siRNA-transfected cells for the same time point in the same experiment. The differently shaped symbols represent five independent experiments. Paired *t*-tests were used to calculate *p*-values. **(C)** The percent of cells that had formed a cSMAC, defined as > 90% of the total Ag fluorescence intensity being contained in one or two clusters, is graphed. The different symbols represent independent experiments. Paired *t*-tests were used to calculate *p*-values. **(D)** The total fluorescence intensity of pCD79 that was present in clusters at the B cell-APC contact site was quantified for each B cell. The left panel shows representative data from one experiment. Each dot is one cell. *n* > 31 cells per condition. The median (blue line) and interquartile ranges (black box) are shown. The Mann-Whitney *U*-test was used to calculate *p*-values. The right panel shows the results from five independent experiments, presented as in **(B)**, with n > 30 cells per condition in each experiment. Each symbol represents a single experiment in which the median pCD79 fluorescence intensity for GMFγ-depleted cells is expressed as a percent of the corresponding median value for the control cells. Paired *t*-tests were used to calculate *p*-values. **(E)** For each B cell represented in **(D)**, the total fluorescence intensity of clustered pCD79 was divided by the total fluorescence intensity of the clustered mHEL-HaloTag Ag. The median (blue line) and interquartile ranges (black box) are shown. The data are presented as in **(B**,**D)**. ****p < 0.0001; ***p < 0.001; **p < 0.01; *p ≤ 0.05; ns, not significant (*p* > 0.05).

**FIGURE 6:**
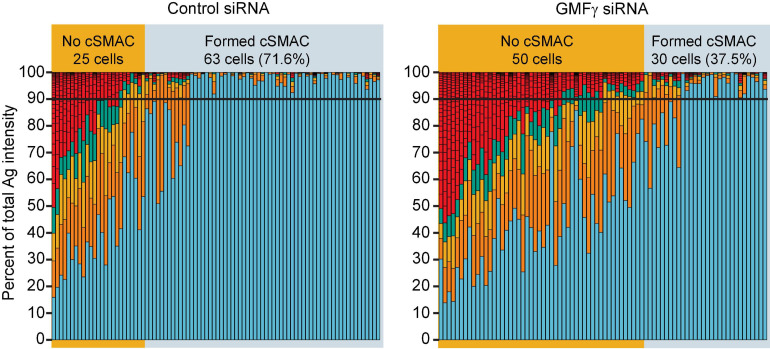
Depleting GMFγ reduces the percent of cells that form a cSMAC. Raji D1.3 B cells that had been transfected with control siRNA or GMFγ siRNA were added to mHEL-HaloTag-expressing COS-7 APCs. The cells were fixed after 30 min and the B cell-APC interface was imaged by spinning disk microscopy. The stacked bar plots show the fraction of the total Ag fluorescence intensity in individual clusters. Each bar represents one cell and each colored segment within a bar represents a single Ag cluster. The size of the colored segment is proportional to the fraction of the cell’s total Ag fluorescence intensity that is contained within that cluster. Cells in which > 90% of the total Ag fluorescence intensity (black horizontal lines) was contained in 1–2 clusters were deemed to have formed a cSMAC. The data are from one of the 5 independent experiments that are compiled in Figure 5C.

We found that depleting GMFγ did not affect the formation of BCR-Ag microclusters but reduced their coalescence into a cSMAC. Both control siRNA- and GMFγ siRNA-transfected Raji D1.3 B cells rapidly formed BCR-Ag microclusters throughout the B cell-APC contact site and, on average, gathered similar amounts of Ag into clusters (Figures 5A,B). Over time, the BCR microclusters in the majority of the control cells coalesced to form a large central cluster. In contrast, a greater number of the GMFγ siRNA-transfected cells failed to form a large central cluster and the Ag fluorescence was instead distributed among multiple small clusters. This difference in microcluster coalescence can be seen by comparing the Ag clusters in the images of control vs. GMFγ siRNA-transfected cells at the 15 min time point in Figure 5A. To more rigorously compare the extent of microcluster coalescence in different cell populations, we applied a quantitative analysis that we developed previously (Bolger-Munro et al., 2019). We determine the amount of Ag fluorescence associated with each discrete Ag cluster on an individual B cell and define cSMAC formation as > 90% of the clustered Ag fluorescence being contained in 1 or 2 clusters. This analysis showed that when control siRNA-transfected cells were added to APCs for 30 min, ∼60% of the cells in 5 independent experiments formed a cSMAC (Figure 5C). In contrast, when GMFγ was depleted, only ∼40% of the cells formed a cSMAC after 30 min (Figure 5C). Data from a representative experiment are presented in Figure 6, which shows the distribution of Ag fluorescence among clusters on individual cells and depicts the number of clusters required to contain > 90% of the Ag fluorescence. This analysis showed that a greater percent of control siRNA-transfected Raji D1.3 B cells formed cSMACs, i.e., gathered > 90% of the Ag fluorescence into one or two clusters, compared to the GMFγ siRNA-transfected cells. Thus, the progressive coalescence of BCR-Ag microclusters that leads to cSMAC formation is reduced when GMFγ is depleted.

The growth and coalescence of BCR-Ag microclusters, as well as their centripetal movement, amplify microcluster-based BCR signaling (Ketchum et al., 2014; Bolger-Munro et al., 2019). To determine whether GMFγ contributes to this signal amplification, we first quantified for each B cell the total amount of pCD79 fluorescence present in clusters at the B cell-APC interface. When GMFγ was depleted from Raji D1.3 cells, the amount of clustered pCD79 per cell was significantly reduced, compared to control siRNA-transfected cells (Figure 5D). This reduction in APC-induced BCR signaling was especially pronounced at the earliest time points, i.e., at 3, 5, and 10 min after adding the B cells to the APCs. In five independent experiments, the median pCD79 levels at the 3 min and 10 time points were reduced by more than 50% when GMFγ was depleted. Hence, GMFγ is a positive regulator of microcluster-based BCR signaling at the immune synapse. For each B cell, we also divided the total amount of pCD79 fluorescence present in clusters at the B cell-APC interface by the total amount of Ag fluorescence in clusters. This reflects the amount of BCR signaling generated per unit of Ag that has been gathered into microclusters, a ratio that we define as “signal amplification.” When GMFγ was depleted, the magnitude of this BCR signal amplification was significantly reduced at the 3, 5, and 10 min time points (Figure 5E). Thus, GMFγ is a positive regulator of BCR signaling amplification, which is associated with the actin-dependent centripetal movement and coalescence of BCR-Ag microclusters.

**FIGURE 7:**
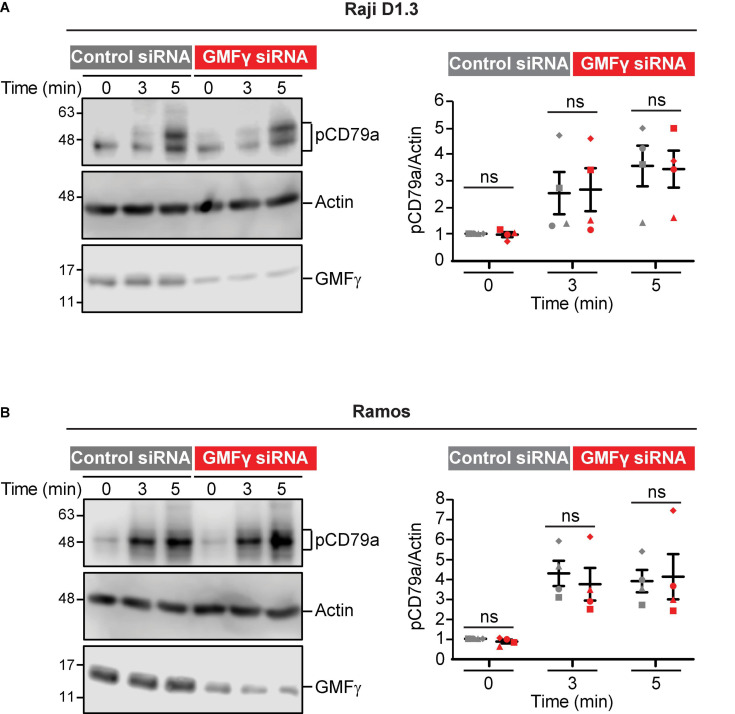
Depleting GMFγ does not impair CD79 phosphorylation induced by soluble anti-Ig antibodies. Cells were transfected with either control siRNA or GMFγ siRNA. **(A)** Raji D1.3 B cells were stimulated with 20 μg/mL goat anti-mouse IgM to initiate signaling through the D1.3 BCR. **(B)** Ramos B cells were stimulated with donkey anti-human IgM. Cell lysates were analyzed by immunoblotting with antibodies that recognize the phosphorylated CD79a ITAM (pCD79a), actin (loading control), or GMFγ. The left panels show a representative experiment. The right panels show results from 4 independent experiments. For each sample, the pCD79a band intensity was divided by the corresponding actin band intensity (loading control). The resulting ratios were normalized to that for the 0 min control siRNA cell sample (defined as 1.0) in the same experiment. On the graph, each of the four experiments is indicated by a different symbol. The bars show the mean ± SEM. Paired *t*-tests were used to calculate *p*-values. ns, not significant (*p* > 0.05).

In contrast to APC-bound Ags, where Ag binding occurs only at a polarized cell-cell contact site, BCR signaling in response to uniformly distributed soluble BCR ligands is much less dependent on actin dynamics and organization (Bolger-Munro et al., 2019). Consistent with this, we found that reducing the levels of GMFγ in Raji D1.3 B cells had no effect on CD79 phosphorylation in response to soluble anti-Ig antibodies (Figure 7A). Similar results were obtained in Ramos B cells (Figure 7B). Thus, GMFγ enhances BCR signaling in response to APC-bound Ags (Figure 5) but appears to be dispensable for responses to soluble BCR ligands. This indicates that GMFγ is not a direct regulator of BCR signaling but instead regulates actin dynamics that amplify BCR signaling responses to spatially restricted Ag arrays, such as those on the surface of APCs.

## Discussion

Actin-disassembly proteins play a central role in remodeling actin networks and also fuel actin polymerization by liberating actin monomers from filaments. Hence, the complex changes in actin dynamics and architecture that drive immune synapse formation are likely to depend on the actions of actin-disassembly proteins. In this study we show that GMFγ supports the ability of B cells to spread across anti-Ig- and Ag-coated surfaces, enhances cSMAC formation at the B cell-APC contact site, and amplifies APC-induced BCR signaling. The lamellipodial protrusion that drives B cell spreading on rigid substrates, as well as the coalescence of BCR microclusters into a cSMAC in response to APC-bound Ags, is driven by Arp2/3 complex-dependent actin polymerization. Hence, GMFγ is a positive regulator of BCR-induced actin remodeling that works in concert with the Arp2/3 complex.

*In vitro* biochemical studies had suggested that GMFγ can antagonize Arp2/3 complex function. At high concentrations, which may be non-physiological, GMFγ can bind to the Arp2/3 complex and cause a conformational change that prevents the nucleation of new actin filaments that branch off from the mother filament (Sokolova et al., 2017). However, at lower concentrations, GMFγ primarily causes debranching, releasing both the Arp2/3 complex and the daughter filament from the mother filament (Gandhi et al., 2010; Ydenberg et al., 2013). The released daughter filaments would have exposed pointed ends, which are sites of actin filament disassembly (Pollard, 2016; Goode et al., 2018). The resulting actin monomers can be converted to the polymerization-competent ATP-bound form by profilin (Kotila et al., 2018). Profilin-actin complexes can then bind to membrane-associated nucleation promoting factors such as WASp, which can interact with actin filament-associated Arp2/3 complexes. This allows the direct delivery of actin monomers to the Arp2/3 complex and their assembly into new branches (Mullins et al., 2018). In addition to providing actin monomers to fuel Arp2/3 complex-dependent actin polymerization, GMFγ-mediated debranching releases Arp2/3 complexes from existing filaments, allowing them to nucleate new actin branches and promote actin polymerization. Because the amount of nucleation-competent ATP-bound Arp2/3 complexes may be limiting within cells, recycling of Arp2/3 complexes is thought to be important for the sustained assembly of dendritic actin networks and the formation of lamellipodia. The role of GMFγ in promoting Arp2/3 complex release and recycling within lamellipodia is supported by studies in fibroblasts showing that the amount of Arp2/3 complex present in lamellipodia is increased when the related GMFβ protein is depleted and decreased when GMFβ is overexpressed (Haynes et al., 2015). Hence, instead of opposing Arp2/3 complex function, GMF proteins may be important for sustaining Arp2/3 complex-dependent processes.

Our findings support the idea that GMFγ works in concert with the Arp2/3 complex in B cells. The radial spreading of B cells on immobilized Ags, which is driven by Arp2/3 complex-dependent actin polymerization, was significantly impaired when GMFγ was depleted. The difference in the substrate contact area between control and GMFγ-depleted B cells becomes progressively larger over time, suggesting that the GMFγ-depleted cells may run out of polymerization-competent actin monomers or free Arp2/3 complexes. In other cell types, GMFγ is also a positive regulator of processes that require actin polymerization such as membrane ruffling, leading edge protrusion, and cell migration (Ikeda et al., 2006; Aerbajinai et al., 2011, 2016; Lippert and Wilkins, 2012; Poukkula et al., 2014). GMFγ preferentially releases older actin branches where the Arp2/3-bound ATP has been hydrolyzed to ADP (Boczkowska et al., 2013; Pandit et al., 2020). This fits with the actin treadmilling model for lamellipodial protrusion in which older segments of the actin network that are further from the membrane are disassembled into monomers, which can then be recycled and used for Arp2/3 complex-nucleated actin polymerization at the plasma membrane (Carlier and Shekhar, 2017). Consistent with this model, when B cells spread on immobilized BCR ligands and formed broad lamellipodia, we found that GMFγ abutted, and perhaps overlapped somewhat, with the inner face of the peripheral actin ring. Importantly, GMFγ was present at much lower amounts in the outer portion of this leading edge than elsewhere in the cell. This may allow the Arp2/3 complex to nucleate actin polymerization at the plasma membrane, and drive the outward expansion of lamellipodia, with less opposition from GMFγ.

We have previously shown that Arp2/3 complex-dependent actin polymerization generates actin retrograde flow at the cell periphery, which drives the initial centralization of BCR-Ag microclusters and promotes their coalescence (Bolger-Munro et al., 2019). This amplifies microcluster-based BCR signaling and leads to cSMAC formation. We found that depleting GMFγ significantly decreased the velocity of actin retrograde flow and reduced cSMAC formation. By providing a source of polymerization-competent actin monomers and recycled Arp2/3 complexes, GMFγ may help sustain the peripheral actin polymerization that generates actin retrograde flow and drives the progressive coalescence of BCR-Ag microclusters. The formation of larger BCR clusters enhances BCR signaling (Ketchum et al., 2014) and we showed that GMFγ helps amplify BCR signaling at the B cell-APC contact site, as assessed by the amount of pCD79 per unit of clustered Ag. The contribution of GMFγ to actin retrograde flow at the immune synapse may also help amplify microcluster-based BCR signaling. The BCR is a mechanosensitive receptor (Shaheen et al., 2019) and the retrograde flow of actin structures could exert force on BCR microclusters that amplify BCR signaling. Overall, our findings are consistent with the idea that GMFγ works in concert with the Arp2/3 complex to enhance BCR microcluster coalescence and microcluster-based BCR signaling at the B cell-APC immune synapse.

Because Ags larger than ∼70 kDa cannot freely diffuse into the B cell follicles within lymphoid organs, APCs play a critical role in B cell responses to larger Ags such as microbial pathogens (Batista and Harwood, 2009; Cyster, 2010; Heesters et al., 2016). In the lymph nodes, a specialized subpopulation of macrophages that line the subcapsular sinus utilizes cell surface lectins, complement receptors, and Fc receptors to capture bacteria, viruses, and immune complexes that are present in the incoming lymphatic fluid. These subcapsular sinus macrophages are in contact with the B cell follicle and can transport the captured Ag from its lumenal side to the side facing the B cell follicle, where they present the Ag to B cells. The Ag can also be shuttled from the subcapsular sinus macrophage to follicular dendritic cells in the center of the B cell follicle (Phan et al., 2007, 2009). Follicular dendritic cells are specialized APCs that produce chemokines that attract B cells (Heesters et al., 2014). B cell interactions with Ag-bearing follicular dendritic cells are essential for the germinal center response that leads to affinity maturation of the antibody response and Ig class switching (Heesters et al., 2016). The important physiological role of APC-mediated B cell activation provides a strong rationale for identifying the cytoskeletal regulators that amplify BCR signaling at the immune synapse, a process that allows small amounts of APC-bound Ag to activate B cells. Indeed, Batista and colleagues showed that 100-fold lower concentrations of Ag are required for B cell activation when the Ag is presented on an APC than when it is in a soluble form (Batista et al., 2001). The unique role of actin dynamics in APC-induced B cell activation is highlighted by the observation that the Arp2/3 complex (Bolger-Munro et al., 2019), the Cdc42 activator DOCK8 (Randall et al., 2009; Sun et al., 2018), and CD19 (Depoil et al., 2008), a transmembrane protein that recruits the Rac/Cdc42 activator Vav, are important for B cell responses to membrane-bound Ags but are all dispensable for responses to soluble Ag. Consistent with this, we showed that reducing GMFγ levels impaired BCR signaling in response to APC-bound Ag but not soluble Ag.

BCR signaling is critical for B cell development and the magnitude of BCR signaling determines whether Ag encounter results in B cell activation, as opposed to the deletion or silencing of potentially self-reactive B cells (Cashman et al., 2019; Meffre and O’Connor, 2019; Tan et al., 2019). Autoimmunity can result from excessive BCR signaling as well as failures to eliminate self-reactive B cells in which BCR signaling is reduced. Indeed, many immunodeficiency syndromes in which immune responses to Ag challenge are impaired are associated with autoimmunity and the production of self-reactive antibodies (Cashman et al., 2019; Meffre and O’Connor, 2019; Tan et al., 2019). By amplifying microcluster-based BCR signaling in response to APC-bound Ags, actin remodeling may modulate the Ag density thresholds that determine whether a B cell-APC encounter leads to B cell activation or tolerance. Hence, it is not surprising that mutations in a number of actin-regulatory proteins lead to actinopathies, immune dysregulation syndromes characterized by autoimmunity as well as immune deficiency that results in recurring microbial infections (Sprenkeler et al., 2020). Reduced BCR signaling in response to cell-associated Ags could compromise the ability to delete self-reactive B cells, allowing them to be activated under certain conditions. Conversely, B cell responses to bacteria and viruses are particularly dependent on APCs and impaired BCR signaling to microbial Ags that are captured by APCs may result in deficient immunity to infection. Indeed, the ability of APC-bound Ags to induce B cell proliferation and activation is reduced when the Arp2/3 complex is inhibited (Bolger-Munro et al., 2019). This suggests that actin-regulatory proteins that work in concert with the Arp2/3 complex, such as GMFγ, may also influence the threshold for B cell activation and tolerance.

There are two GMF genes in vertebrate genomes, GMFβ and GMFγ. The two GMF proteins are 82% identical in amino acid sequence, have similar 3-dimensional structures, and both can mediate actin debranching (Goode et al., 2018). Like GMFγ, GMFβ also regulates lamellipodial actin dynamics, at least in fibroblasts (Haynes et al., 2015). RNA-Seq data suggest that both GMFs are expressed in B cells^4^. Whether GMFβ also contributes to BCR-induced actin remodeling, B cell spreading, cSMAC formation, or APC-induced BCR signaling remains to be determined. Nevertheless, we showed that selectively reducing the levels of GMFγ was sufficient to impair all of these actin-dependent processes. The debranching activity of GMF proteins may also be regulated by phosphorylation. *In vitro*, recombinant GMFγ with a phosphomimetic S2E mutation exhibits a greatly reduced affinity for ADP-bound Arp2/3 complex (Boczkowska et al., 2013). This suggests that there could be dynamic regulation of GMF-mediated debranching that is coordinated with the initiation and termination of lamellipodial protrusion or other actin-dependent processes.

GMFγ is one of many actin-disassembly proteins that contribute to the spatiotemporal regulation of actin dynamics and remodeling. Cofilin and related actin-severing proteins are essential for actin network remodeling (Kadzik et al., 2020). Like GMFγ, cofilin preferentially disassembles older portions of peripheral actin networks that are further from the plasma membrane (Bernstein and Bamburg, 2010). In lamellipodia, the actions of the Arp2/3 complex and cofilin are tightly coupled with cofilin-dependent recycling of actin monomers and Arp2/3 complexes sustaining actin treadmilling (Carlier and Shekhar, 2017). We have shown that cofilin and its co-factor Wdr1 are critical regulators of actin dynamics and organization in B cells, and are important for B cell spreading as well as the actin-dependent amplification of BCR signaling at the immune synapse (Freeman et al., 2011; Bolger-Munro et al., 2021). In contrast to cofilin, capping proteins and their interactors regulate actin monomer depolymerization at the barbed ends of filaments (Edwards et al., 2014). In particular, twinfilin substantially increases the rate of barbed end depolymerization (Hilton et al., 2018) and depleting twinfilin decreases actin turnover dynamics at the leading edge (Hakala et al., 2021). Finally, GMFγ, coronins, and arpin regulate actin dynamics by targeting the Arp2/3 complex (Sokolova et al., 2017). These proteins bind to different sites on the Arp2/3 complex and, *in vitro*, they all induce the Arp2/3 complex to assume an “open” conformation that cannot nucleate branched actin polymerization. This suggests that they may have partly overlapping functions. GMFγ, coronins, and arpin can also act in additive or synergistic manners. However, only GMFγ catalyzes actin debranching by binding to the Arp2/3 complex and causing the dissociation of the Arp2/3 complex that bridges the daughter filament to the mother filament (Sokolova et al., 2017). Together, this network of Arp2/3 complex regulators may enable precise spatial and temporal regulation of branched actin polymerization and the architecture of dendritic actin networks.

Because GMFγ, coronins, and arpin may regulate the Arp2/3 complex in a cooperative manner, it is not surprising that depleting GMFγ has less dramatic effects on B cell spreading and B cell responses to APCs than what we observed when the Arp2/3 complex was depleted or inhibited (Bolger-Munro et al., 2019). The role of arpin in B cells has not been examined. Coronins are multifunctional proteins that regulate multiple aspects of immune cell function (Tokarz-Deptula et al., 2016). Coronin 1 is important for T cell homeostasis as well as TCR- and BCR-induced Ca^2+^ responses (Mueller et al., 2008; Combaluzier et al., 2009; Siegmund et al., 2011). However, the role of coronin 1 in actin-dependent processes in B cells remains unexplored. Because actin dynamics amplify APC-induced BCR signaling, dissecting the contributions of different actin-disassembly proteins may provide new insights into the regulation of B cell activation.

## Data Availability Statement

The raw data supporting the conclusions of this article will be made available by the authors, without undue reservation.

## Author Contributions

MB-M and ND conceived and designed the experiments. ND, KC, LA, and MB-M performed the experiments. ND, MB-M, KC, and MG analyzed the results. ND, MB-M, and MG wrote the manuscript with input from KC. MG was the principal investigator of the study. All authors contributed to the article and approved the submitted version.

## Conflict of Interest

The authors declare that the research was conducted in the absence of any commercial or financial relationships that could be construed as a potential conflict of interest.

## Footnotes

1 http://questpharma.u-strasbg.fr/html/radial-profile-ext.html

2 Baggethun P. 2009. Image analysis: radial profile plot https://www.researchgate.net/publication/317704672_Image_analysis_Radial_profile_plot

3 https://github.com/mbolgermunro/FIJImacros/blob/master/APC_analyser_MBM.ijm

4 http://rstats.immgen.org/Skyline/skyline.html

## Abbreviations

Ag: antigen
APC: antigen-presenting cell
Arp: actin-related protein
BCR: B cell antigen receptor
BSA: bovine serum albumin
cSMAC: central supramolecular activation cluster
F-actin: filamentous actin
FCS: fetal calf serum
GMF: glia maturation factor
HEL: hen egg lysozyme
Ig: immunoglobulin
ITAM: immunoreceptor tyrosine-based activation motif
mHBS: modified HEPES-buffered saline
pCD79: phosphorylated CD79
PFA: paraformaldehyde
TIRF: total internal reflection fluorescence
WASp: Wiskott-Aldrich Syndrome protein.
