# Supplementary material for: The Actin-Disassembly Protein Glia Maturation Factor γ Enhances Actin Remodeling and B Cell Antigen Receptor Signaling at the Immune Synapse
Source: Front Cell Dev Biol. 2021 Jul 9;9:647063. doi: 10.3389/fcell.2021.647063 (PMC8318000; doi:10.3389/fcell.2021.647063)
Supplement: Supplementary file 1 [file Data_Sheet_1.pdf]

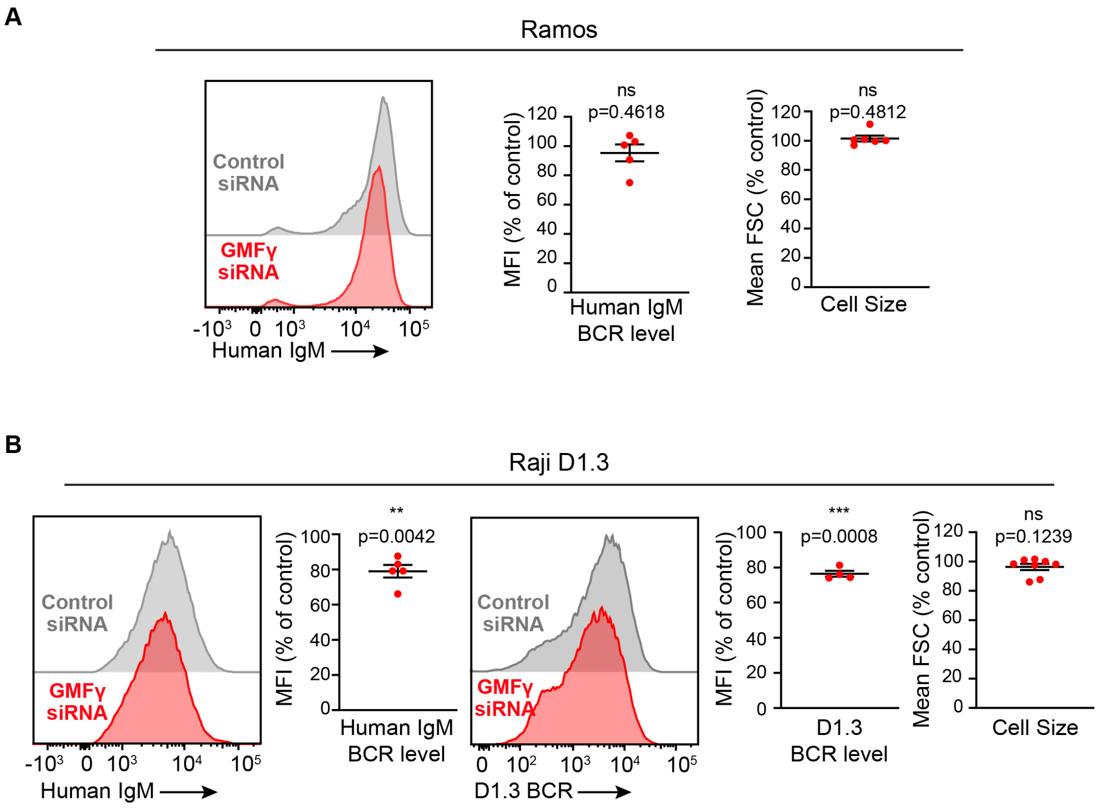

SUPPLEMENTARY FIGURE 1 | Effects of GMF-gamma depletion on BCR cell surface levels and cell size.

Ramos **(A)** or Raji D1.3 B cells **(B)** were transfected with control non-targeting siRNA or GMFγ siRNA and then cultured for 48-72 h. The cells were stained with antibodies to human IgM to detect the endogenous BCR or with anti-mouse IgM to detect the D1.3 BCR on Raji D1.3 B cells. Flow cytometry was used to quantify cell surface IgM levels as well as forward scatter, a measure of cell size. Histograms from representative experiments are shown. The graphs show the cell surface BCR levels (mean fluorescence intensity) or cell size (mean forward scatter) for the GMFγ siRNA-transfected cells, expressed as a percent of that for the control siRNA-transfected cells in the same experiment. Each dot is an independent experiment. Paired t-tests were used to calculate p-values.

MOVIE CAPTIONS

Movie 1 | Peripheral actin dynamics in control siRNA-transfected B cells plated on immobilized anti-IgM. Raji D1.3 B cells that had been co-transfected with F-tractin-GFP cDNA and control siRNA were added to anti-IgM-coated coverslips. After allowing the cells to spread for 5 min, the cell-substrate contact site was imaged using TIRF microscopy. Images were then acquired every 2 s for 15 min. Video playback is 60 frames per second (120X real time). See **Figure 4**.

Movie 2 | Peripheral actin dynamics in GMFγ siRNA-transfected B cells plated on immobilized anti-IgM. Raji D1.3 B cells that had been co-transfected with F-tractin-GFP cDNA and GMFγ siRNA were added to anti-IgM-coated coverslips. After allowing the cells to spread for 5 min, the cell-substrate contact site was imaged using TIRF microscopy. Images were then acquired every 2 s for 15 min. Video playback is 60 frames per second (120X real time). See **Figure 4**.
